# Genome-wide association analysis reveals loci associated with resistance against *Piscirickettsia salmonis* in two Atlantic salmon (*Salmo salar* L.) chromosomes

**DOI:** 10.1186/s12864-015-2038-7

**Published:** 2015-10-24

**Authors:** Katharina Correa, Jean P. Lhorente, María E. López, Liane Bassini, Sudhir Naswa, Nader Deeb, Alex Di Genova, Alejandro Maass, William S. Davidson, José M. Yáñez

**Affiliations:** Facultad de Ciencias Veterinarias y Pecuarias, Universidad de Chile, Av Santa Rosa 11735, Santiago, Chile; Aquainnovo, Talca 60, Puerto Montt, Chile; Facultad de Ciencias Agronómicas, Universidad de Chile, Av Santa Rosa 11315, Santiago, Chile; Genus plc, 100 Bluegrass Commons Blvd. Suite 2200, Hendersonville, TN 37075 USA; Laboratory of Bioinformatics and Mathematics of the Genome, Center for Mathematical Modeling (UMI 2807 CNRS) and Center for Genome Regulation, Universidad de Chile, Beauchef 851, Santiago, Chile; Department of Molecular Biology and Biochemistry, Simon Fraser University, 8888 University Drive, Burnaby, BC Canada

**Keywords:** Salmon Rickettsial Syndrome, Genome Wide Association Analysis, Pathogen resistance, Atlantic salmon, Single Nucleotide Polymorphism

## Abstract

**Background:**

*Pisciricketssia salmonis* is the causal agent of Salmon Rickettsial Syndrome (SRS), which affects salmon species and causes severe economic losses. Selective breeding for disease resistance represents one approach for controlling SRS in farmed Atlantic salmon. Knowledge concerning the architecture of the resistance trait is needed before deciding on the most appropriate approach to enhance artificial selection for *P. salmonis* resistance in Atlantic salmon. The purpose of the study was to dissect the genetic variation in the resistance to this pathogen in Atlantic salmon.

**Methods:**

2,601 Atlantic salmon smolts were experimentally challenged against *P. salmonis* by means of intra-peritoneal injection. These smolts were the progeny of 40 sires and 118 dams from a Chilean breeding population. Mortalities were recorded daily and the experiment ended at day 40 post-inoculation. Fish were genotyped using a 50K Affymetrix® Axiom® myDesign^TM^ Single Nucleotide Polymorphism (SNP) Genotyping Array. A Genome Wide Association Analysis was performed on data from the challenged fish. Linear regression and logistic regression models were tested.

**Results:**

Genome Wide Association Analysis indicated that resistance to *P. salmonis* is a moderately polygenic trait. There were five SNPs in chromosomes Ssa01 and Ssa17 significantly associated with the traits analysed. The proportion of the phenotypic variance explained by each marker is small, ranging from 0.007 to 0.045. Candidate genes including interleukin receptors and fucosyltransferase have been found to be physically linked with these genetic markers and may play an important role in the differential immune response against this pathogen.

**Conclusions:**

Due to the small amount of variance explained by each significant marker we conclude that genetic resistance to this pathogen can be more efficiently improved with the implementation of genetic evaluations incorporating genotype information from a dense SNP array.

**Electronic supplementary material:**

The online version of this article (doi:10.1186/s12864-015-2038-7) contains supplementary material, which is available to authorized users.

## Background

The control of infectious diseases is a prime concern in salmon farming due to severe economic losses, reduced animal welfare and challenges to the sustainability of the industry [1, 2]. *Piscirickettsia salmonis* is an intracellular bacterium, causing Salmon Rickettsial Syndrome (SRS) [3] in salmon species and is considered one of the major pathogens of the salmon farming industry. During *P. salmonis* outbreaks fish exhibit pale gills, abdominal swelling and haemorrhages on the base of fins [4]. Infected fish often have skin lesions [5, 6]. Internally, serosanguinous ascites and swollen kidneys, livers and spleens are common. *P. salmonis* is horizontally transmitted through gills or skin [7]. This pathogen has evolved over time, being insidious with each outbreak and becoming refractory to treatments [4]. Antibiotic use may inhibit the growth of the pathogen, but treatments have been unsuccessful in stopping disease outbreaks [8]. Commercial vaccines have also not proven to be as efficient as needed [4, 9, 10]. In Chile alone, *P. salmonis* is responsible for the 74 % of the infectious-related deaths in Atlantic salmon [11] resulting in economic losses estimated at US $100 million [12].

Disease resistance has become an increasingly important trait of interest included in the genetic programs of aquatic species [1, 13, 14]. Selective breeding for disease resistance in aquaculture species commonly includes survival information from experimental infection of full-sibs of the breeding candidates [1, 13, 14]. The identification of the most resistant families is performed using the information obtained from challenge results, which can be used to identify the genomic regions (i.e. quantitative trait loci, QTL) involved in the genetic variation of these traits [1, 13, 14]. Selection for disease resistance traits is based on estimated breeding values (EBVs) calculated using phenotypic records and pedigree. Previous studies have estimated low to moderate heritabilities (proportion of the phenotypic variance that is accounted for by the additive genetic variance), ranging between 0.11 and 0.41 for resistance to *P. salmonis* [15, 16] in the same commercial population of Atlantic salmon used in the present study; indicating a potential for selective breeding for *P. salmonis* resistance in this population.

The availability of molecular tools like dense panels of SNPs (Single Nucleotide Polymorphisms) in domesticated species has led to the search for genomic regions and causal mutations underlying variation in complex traits through the use of linkage and association mapping [17–19]. High-density SNP panels have recently been developed for Atlantic salmon [20, 21]. In Genome-Wide Association Studies (GWAS or association mapping) a group of individuals are phenotyped and then genotyped using a large number of SNPs, in order to detect statistical association between a marker and a trait of interest [22]. Data from GWAS experiments are commonly analyzed testing one SNP at a time using linear models [17]. Two limitations of this approach are that it does not account for epistasis effects (interaction between markers) and adjustments are needed to control the false positive rate due to the multiple testing problem

The associated markers can be used to accelerate the genetic progress by means of Marker Assisted Selection (MAS), if the total variance explained by significant markers is high [23]. MAS can use a causative mutation that has been identified in a gene or regulatory region that has a major effect over the trait; or can use SNPs that are in Linkage Disequilibrium (LD) with a QTL to increase the response to selection [17]. Information from SNP panels can be used for the evaluation of breeding candidates through Genomic Selection (GS). In GS, sibs of the selection candidates are phenotyped and genotyped, while the selection candidates are only genotyped. The sibs of the selection candidates are utilized to predict the effect of each marker, and genomic EBVs (GEBVs) are calculated for the candidates using the information of all genotyped SNPs [24]. Knowledge concerning the architecture of the resistance trait is needed before deciding on the most appropriate approach to enhance artificial selection for *P. salmonis* resistance in Atlantic salmon, (i.e. MAS or GS).

Here we present the first Genome Wide Association Analysis performed on Atlantic salmon to dissect the genetic architecture of resistance to *P. salmonis* using a 50 K SNP array in 2601 fish.

## Methods

### Fish material and records

The origin of the population used in the present study is a Chilean salmon strain derived from the Irish strain Fanad-Mowi (originally from Norway) [25, 26]. Fish from the Fanad-Mowi strain were introduced for farming purposes to Chile during the 1990’s through commercial agreements. In 1997, a breeding program for Atlantic salmon was started by the company AquaChile (Puerto Montt, Chile), aimed at improving economically relevant traits including growth rate, carcass quality traits and late maturation. The base population of the breeding nucleus was established mainly with fish from the Fanad-Mowi strain. More specifically, the population used in the present study corresponds to the year-class 2010, which already has four generations of selective breeding in Chilean farming conditions. Two thousand six hundred one Atlantic salmon smolts weighing on average 274.8 g (SD = 90.6 g) were experimentally challenged against *P. salmonis* as described previously [15]. Briefly, infection was induced through intra-peritoneal (IP) injection with 0.2 ml of a LD50 inoculum of *P. salmonis*. Post-injection, infected fish were distributed in three different tanks with salt water (31 ppt). These smolts were the same individuals used in a previous study [15] and were the progeny of 40 sires and 118 dams from the breeding population of Salmones Chaicas, X Region, Chile. Each individual was identified using a PIT (Passive Integrated Transponder) tag, and a similar number of fish from each of the 118 full sib families were placed in each tank. The average number of fish per family was 22, ranging from 9 to 24. In order to determine the specific response to *P. salmonis* infection, the absence of other pathogens is required. Fish were found to be negative for Infectious Salmon Anemia virus, Infectious Pancreatic Necrosis virus and *Renibacterium salmoninarum* by RT-PCR and negative for *Flavobacterium spp.* by culture. Mortalities were recorded daily and all survivors were anesthetized and euthanized at day 40. Final body weight was recorded at the day of death for every dead fish or at the end of the challenge for survivors. Fin samples were taken from all fish and preserved in ethanol at −80 ° C until DNA extraction. The challenge test and sampling procedures were approved by The Comité de Bioética Animal, Facultad de Ciencias Veterinarias y Pecuarias, Universidad de Chile (Certificate N° 08–2015).

### Genotyping

Genomic DNA was extracted from the fin clips from all challenged fish using a commercial kit (DNeasy Blood & Tissue Kit, Qiagen), following the manufacturer’s instructions. Fish were genotyped using a 50 K Affymetrix® Axiom® myDesign^TM^ SNP Genotyping Array designed by AquaInnovo and the University of Chile.

### 50 K genotyping array

The markers included in this array were chosen from a 200 K SNP array previously developed and validated in six commercial populations (five with European Origin and one with North American origin) and two wild populations (one from Europe and another one from North America) [21]. Affymetrix software SNPolisher [27] was used to retain 159,099 Poly-high-resolution (SNPs that form distinct clusters of good resolution and have at least two occurrences of minor allele) [27] and No- minor-homozygote (SNPs forming two distinct clusters and with no occurrences of minor homozygous genotypes) [27] SNPs. SNPs were further filtered based on Mendelian error in genotypes of 14 trios (sire, dam and offspring) and one sire-offspring pair from one commercial population. 137,712 SNPs with less than 1 Mendelian error were selected. SNPs were further selected on the basis of minor allele frequency (MAF) in five commercial populations of European origin. We gave preference to SNPs with higher MAF in the commercial populations of European origin because the target populations which will be used for further implementation of genomic selection and Genome Wide Association Studies have European origin. For this purpose four related European populations were grouped together as Group A and a fifth population was called Group B. SNPs were selected if they met one of the following two criteria: i) MAF > =0.05 in Group A and MAF > =0.1 in Group B or ii) MAF > =0.05 in Group B and MAF > =0.1 in Group A. From 112,241 SNPs that passed the above criteria, 55,591 SNPs were selected so that they are as evenly distributed along the genome as possible. This was done by selecting SNPs from windows of equal size across various linkage groups of the genome. When selecting SNPs from windows, higher preference was given to SNPs with lower Mendelian error. Pearson correlation was used to estimate LD among the SNP pairs belonging to same linkage group. Only one member of each SNP pair with R-square > =0.99 was retained. For each pair higher preference was given to SNPs near the end of the linkage group or having lower Mendelian error. From the remaining 53,998 candidates, 50,000 SNPs were selected by removing a SNP that had a distance of less than 10Kb from its neighbor, so markers will be distributed equally across the genome.

Genotypes were obtained for our samples following the Best Practice Analysis Workflow from Affymetrix [28] and selecting the Poly-high-resolution and the No-minor-homozygote SNPs from SNPolisher [27]. Quality control was performed on the genotypes to filter based on Hardy Weinberg equilibrium (*p* < 1 × 10^−10^), Minor Allele Frequency (>0.001) and call rate for SNPs and samples (>0.95). For the quality control the R statistical software and the GENAbel library [29, 30] were used.

### Trait definition and genome wide association analysis

The resistance phenotypes were defined as the time to death (TD), measured in days with values ranging from 1 to 40, depending on the day the fish died; and as a binary survival (BS), scored as 1 if the fish died during the 40-day challenge and 0 if the fish survived until the end of the trial. Fish weight at the end of the experiment and tank designation were also recorded to be included as covariate and factor within the model, respectively.

Linear regression and logistic regression were used to identify association between SNPs and resistance to *P. salmonis,* for the TD and BS traits, respectively, using the *mlreg* function implemented in GenABEL [29, 30]. Logistic regression was used to account for the binary nature of the BS trait. A genomic kinship matrix was calculated from SNP data using the *gkin* function. The inbreeding coefficient (F) was estimated using PLINK [31] to determine the extent of inbreeding in our samples. Heritability values were also estimated from genotype data, using a linear mixed model implemented on *polygenic* function [29, 30, 32–34]. The general formula used for the linear regression model is as follows:

$$ {Y}_i={\beta}_0+{\beta}_1\ast SNP+{\beta}_2\ast Tk+{\beta}_3\ast W+{e}_i $$

Where **Y**_**i**_ is the phenotypic record (TD); ***β***_***0***_ the intercept; ***β***_***1***_ the effect of each ***SNP; β***_*2*_ is the effect of each tank (**Tk**); ***β***_***3***_ is the effect of the weight (**W**) and ***e***_***i***_ the random residual. The general formula for the logistic regression model for BS is:

$$ \pi (X)=\frac{e^{\beta 0+\beta 1\ast SNP+\beta 2\ast Tk+\beta 3\ast W}}{1+{e}^{\beta_0+{\beta}_1X\beta 0+\beta 1\ast SNP+\beta 2\ast Tk+\beta 3\ast W}} $$

Where *π*_*i*_ is the probability of the random variable being one, ***β***_***0***_ the intercept; ***β***_***1***_ the effect of each ***SNP; β***_*2*_ is the effect of each tank (**Tk**) and ***β***_***3***_ is the effect of the weight (**W**).

The -log_10_ (*p-value*) for each SNP across the genome was plotted to summarize the GWAS results. The significance threshold was determined using False Discovery Rate correction [35]. This correction was performed as follows: (1) we ordered *p* values *p*_*1*_ *≤ p*_*2*_ *≤ … ≤ p*_*k*_ where *k* is the number of markers tested. (2) Starting with the largest *p* value, we found the first individual *p* value (*p*_*i*_) that satisfied: *p*_*i*_ ≤ *i/k* *0.05, where *i* = *i*th observation. (3) The *p*_*i*_ that satisfied the condition above became the critical value for the experiment.

The proportion of the heritability explained by each significant marker was obtained by comparing the heritability estimated with the *polygenic* function and the heritability estimated with the inclusion of the significant SNP genotype as factor [36]. The proportion of the phenotypic variance explained by each marker for both traits was estimated multiplying the heritability by the proportion of heritability explained by each marker. The approximate effect of each marker was obtained from the linear regression analysis performed in GenABEL [29, 30]. The level of linkage disequilibrium between significant markers was determined by calculating *r*^*2*^ statistics using the *r2fast* function implemented in GenABEL [29, 30, 37]. For *r*^*2*^ calculations we used all genotyped samples which passed the QC.

## Results and discussion

**Fig. 1:**
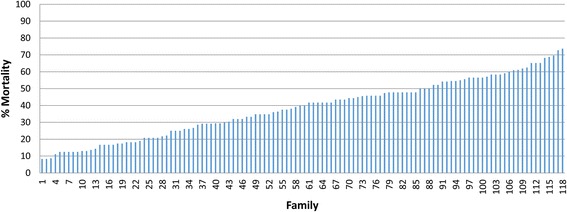
Cumulative mortality by family after *Piscirickettsia salmonis* experimental infection. The average mortality after 40 days of experimental infection against *P. salmonis* is plotted for each of the 118 full-sib families of Atlantic salmon included in the present study

The average mortality per family at the end of the test was 38.4 % (SD = 17.1, minimum = 8.3 %, maximum = 73.7 %) (Fig. 1). We observed considerable variation in the phenotypic survival across families: some families showed more resistance with a low mortality (as low as 8 %), while other families had high mortality (up to 73 %).

Final body weight was recorded as a covariate in the statistical models as previously described [15, 16]. Average final body weight was 326.7 g (SD = 137 g; CV = 42 %). Fish grew during the trial, and the increased SD and CV reflects a higher level of dispersion of final body weight compared with body weight measured at the beginning of the trial (SD = 90.6; CV = 33 %). This is likely due to varying days of death among animals, as some fish died early in the trial (having less time to grow) and some survived the entire trial period.

Forty-eight thousand eight hundred eighty-six SNPs and 2391 samples passed all quality control criteria. Average F was 0.017 (SD = 0.008). Thus, the inbreeding level in this population is considered low and will have a small impact on the power of the association analysis. Furthermore, we accounted for population structure within the sample aimed at decreasing the chances of occurrence of type I and type II errors in the association analysis. The heritability estimated using the genomic information was 0.19 for TD and 0.20 for BS (*p* values < 0.00001) [30], which is consistent with the results obtained in previously reported estimations using pedigree information [15, 16]. In these previous studies when the binary survival (0/1) was analysed using a threshold model, a heritability of 0.24 ± 0.04 was calculated [15]. When a linear model was used to analyse the day of death, a heritability of 0.18 ± 0.03 was estimated [15, 16].

**Fig. 2:**
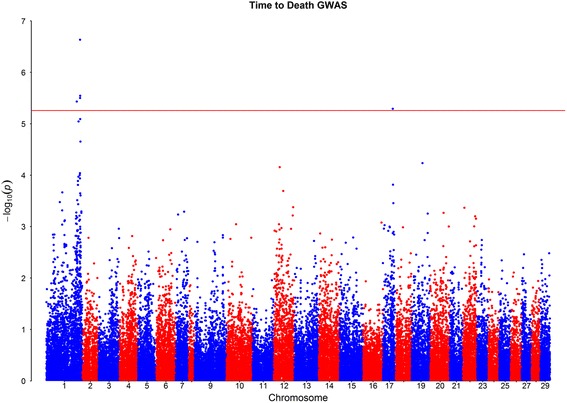
GWAS for *Piscirickettsia salmonis* resistance measured as time to death: Red line indicates False Discovery Rate significance threshold

**Fig. 3:**
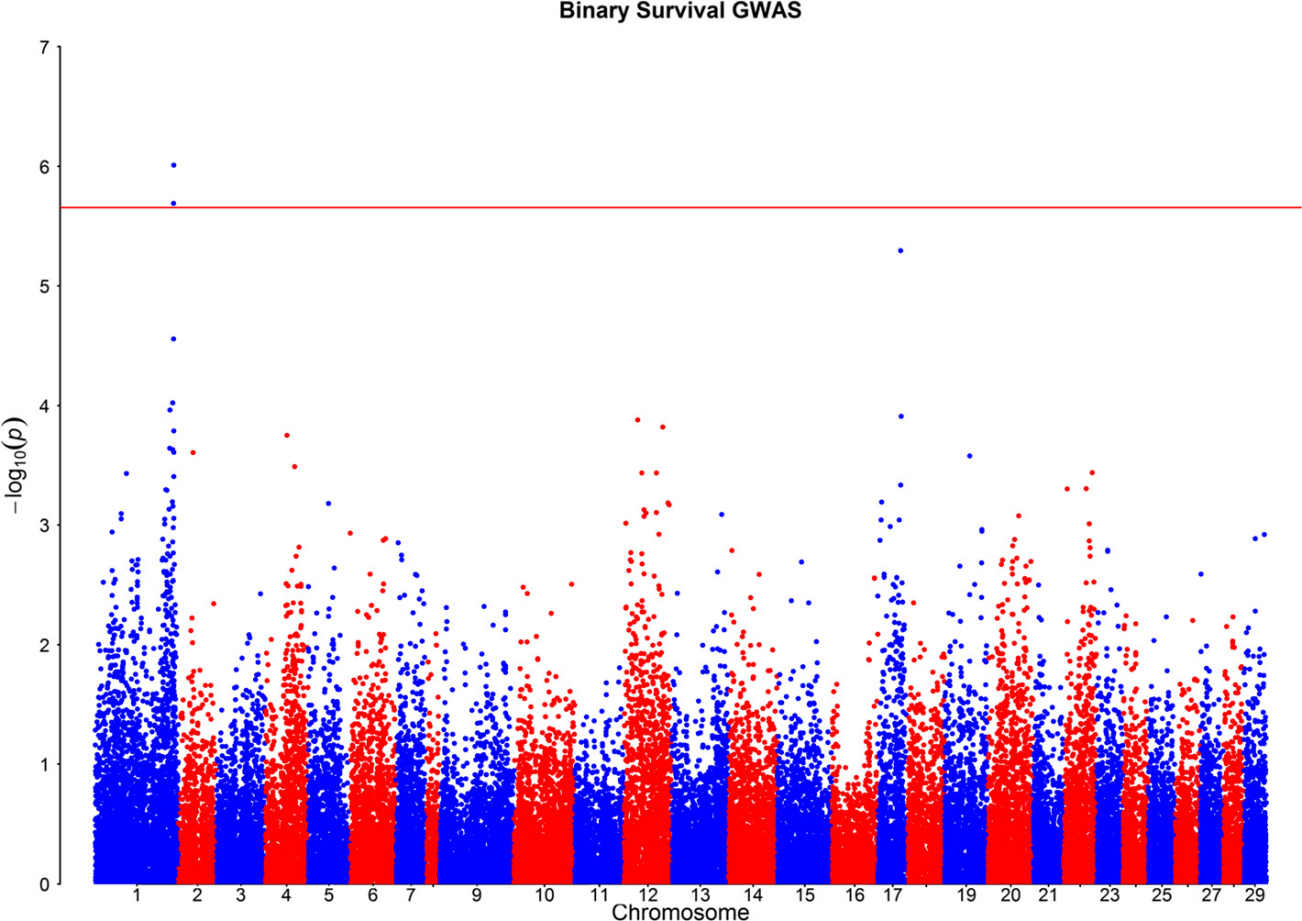
GWAS for *Piscirickettsia salmonis* resistance measured as binary survival: Red line indicates False Discovery Rate significance threshold

The *p* values obtained from the GWAS indicate evidence of significant associations on Atlantic salmon chromosomes Ssa01 and Ssa17 (Figs. 2 and 3).

Results were consistent between models after multiple testing corrections (Table 1). The linear model performed slightly better in terms of detecting significant markers associated with TD than the logistic model for BS. A linear model using TD appears to be a better fit than a logistic model using BS; which leads to more efficient identification of important SNPs. Similarly, a slightly higher accuracy of EBVs was obtained when fitting a linear model to analyse day of death when compared to a threshold model to analyse overall test period survival (binary) for *P. salmonis* resistance in Atlantic salmon [15].

**Table 1:** Significant SNPs found for *Piscirickettsia salmonis* resistance in *S. salar* detected for time to death and binary survival traits

SNP_ID	Chr	Contig	Position (Chr)	Binary survival	Time to death
AQI_UCh-93346943	Ssa01	Jcf1000860129	150,070,839	9.78e-07^a^	2.80e-06^a^
AQI_UCh-93451692	Ssa01	Jcf1000860129	149,409,925	2.04e-06^a^	2.31e-07^a^
AQI_UCh-93323028	Ssa01	Jcf1000860129	149,440,843	2.78e-05	3.15e-06^a^
AQI_UCh-93268476	Ssa17	Jcf1001033219	44,003,022	5.07e-06	5.10e-06^a^
AQI_UCh-93384759	Ssa01	Ccf000000073	134,754,075	0.00050	3.69e-06^a^

^a^Significant after FDR corrections

The logistic regression model revealed two significant SNP associated with BS located in one genomic region of chromosome Ssa01 (AQI_UCh-93346943 and AQI_Uch-93451692). In the linear regression model, five significant SNPs associated with TD were identified, the same two identified by the logistic regression and three additional SNPs. One marker is located in the same genomic region of the afore-mentioned SNPs (AQI_UCh-93323028) and two are located in different regions of chromosomes Ssa01 and Ssa17 (AQI_UCh-93268476 and AQI_UCh-93384759). Two of these markers are significant for both trait definitions and models used. Markers that were significant only for TD were suggestive for BS. The resistance alleles are the minor alleles, with frequencies ranging between 0.24 and 0.31 (Table 2), indicating that there is still potential to increase their frequencies in the breeding population.

**Table 2:** Minor allele frequency of each marker and effect of each allele on the resistance against *Piscirickettsia salmonis*

SNP id	A_1_	A_2_	MAF	A_1_ A_1_	A_1_ A_2_	A_2_ A_2_	Effect (A_2_)
AQI_UCh-93346943	C	A	0.24	34.96	32.77	31.33	−2.15 (0.26)
AQI_UCh-93451692	C	A	0.29	34.64	32.27	29.34	−2.91 (0.32)
AQI_UCh-93323028	G	T	0.31	34.53	32.04	30.24	−2.73 (0.33)
AQI_UCh-93268476	C	T	0.24	35.40	33.50	31.86	−1.89 (0.23)
AQI_UCh-93384759	G	T	0.31	34.70	32.74	29.93	−2.31 (0.28)

A_1_ and A_2_ are the minor and major alleles, respectively. Mean value of the time to death by genotype for each marker. A_1_: high resistance allele. Effect of the A_2_ allele and standard error in brackets

It is interesting to note that three of the identified markers are located in a ~660Kb region (contig Jcf1000860129) in chromosome Ssa01. This region contains several predicted genes [38] and some of them could play an important role in the resistance against *P. salmonis* (Table 3). AQI_UCh-93451692 is located in an intronic region of Alpha-(1,3)-Fucosyltransferase, which is involved in neutrophil and T cell recruitment and lymphocyte trafficking [39–41]. The SNP marker located on chromosome Ssa17 is close to a Metalloprotease, described as up-regulated in Atlantic salmon infected with *P. salmonis* [42]. SNP AQI_UCh-93384759 is located in an intronic region of a predicted gene corresponding to Tenascin, which has been described to generate an effective immune response to bacterial lipopolysaccharide [43]. Other genes like interleukin receptors are located in the genomic regions identified in the GWAS and could be involved in differential susceptibility to *P. salmonis* infection. Some of these genes have been previously identified in gene expression assays of diseased fish [42], and thus these results support the evidence that they may be involved in Atlantic salmon’s response to *P. salmonis.* Further assays like differential expression or sequencing of selected genes and regions in susceptible and resistance fish could lead to a better understanding of the biological response to this pathogen.

**Table 3:** Summary of the locations and functions of candidate genes that could play an important role in the immune response to *P. salmonis* infection and that have been identified in regions surrounding the most significant markers

Gene	Position in contig	Function/comments	Reference
Alpha-1,3-fucosyltransferase 10	jcf1000860129_0-0_ssa01: 2897333..2936162 (− strand)	Neutrophil and T cells recruitment, lymphocyte trafficking.	[39–41]
Interleukin 31 receptor A	jcf1000860129_0-0_ssa01:3283056..3291004 (+ strand)	Involved in IL-31 signaling via activation of STAT-3 and STAT-5. Potentially involved in the development and function of monocytes and macrophages.	[50, 51]
Interleukin 6 signal transducer	jcf1000860129_0-0_ssa01:3294763..3315063 (− strand)	Signal transducer shared by many cytokines, including interleukin 6. This protein functions as a part of the cytokine receptor complex.	[52]
Granulocyte colony-stimulating factor receptor	jcf1000860129_0-0_ssa01:3294763..3315063 (− strand)	Regulation of myelopoiesis, promotion of the survival, proliferation and differentiation of Granulocyte-Macrophages Progenitors. Chemoattractive to immature and mature neutrophils*;* enhances neutrophil anti-microbial functions and neutrophil survival. Can also promote monocytes to differentiate into inflammatory dendritic cells.	[53, 54]
Mitogen-activated protein kinase kinase kinase (MAPKKK)	jcf1000860129_0-0_ssa01:3441205..3459556 (+ strand)	Participates in the activation of MAPK, which is crucial for transcriptional and nontranscriptional responses of the immune system. Upregulated in Atlantic salmon infected with *P. salmonis.*	[42, 55]
Metalloprotease ATP23	jcf1001033219_0-0_ssa17: 1317970..1353399 (− strand)	Matrix metalloproteinase are upregulated in Atlantic salmon infected with *P. salmonis*	[42]
TNF receptor-associated factor 2	ccf1000000073_0-0_ssa01:2138109..2155775 (− strand)	Required for normal antibody isotype switching from IgM to IgG. Regulates activation of NF-kappa-B and JNK and plays a central role in the regulation of cell survival and apoptosis.	[56, 57]

The proportion of the heritability explained by each identified marker ranged between 0.09 and 0.18 for TD and 0.03 and 0.24 for BS (Table 4). The proportion of phenotypic variance explained by each marker was small, ranging from 0.007 to 0.045 for BS and from 0.017 to 0.035 for TD.

**Table 4:** Proportion of heritability and phenotypic variance explained by each marker for time to death and binary survival traits

SNP_ID	Proportion of Heritability (TD)	Proportion of Phenotypic Variance (TD)	Proportion of Heritability (BS)	Proportion of Phenotypic Variance (BS)
AQI_UCh-93346943	0.158	0.030	0.148	0.029
AQI_UCh-93451692	0.184	0.035	0.233	0.045
AQI_UCh-93323028	0.161	0.031	0.143	0.028
AQI_UCh-93268476	0.100	0.019	0.115	0.022
AQI_UCh-93384759	0.091	0.017	0.036	0.007

Markers located in the same contig (Jcf1000860129) exhibit high level of LD, ranging between 0.53 and 0.84 (Table 5). The LD between markers in this contig and the marker located in the other contig (Ccf000000073) in chromosome Ssa01 is also relatively high (0.37-0.65), which is consistent with the physical proximity of both contigs. The marker located on chromosome Ssa17 showed low LD with other markers. The closer the markers are located, the higher the value of r^2^ is to be expected [17]. Due to the high amount of LD present between the markers identified, their effect is likely to be shared.

**Table 5:** Linkage disequilibrium estimates (r^2^) between pairs of markers are shown above the diagonal

	AQI_UCh-93346943	AQI_UCh-93451692	AQI_UCh-93323028	AQI_UCh-93268476	AQI_UCh-93384759
AQI_UCh-93346943	-	0.6670	0.5375	0.0508	0.3770
AQI_UCh-93451692	2380	-	0.8423	0.0984	0.6508
AQI_UCh-93323028	2378	2387	-	0.0863	0.5313
AQI_UCh-93268476	2378	2387	2385	-	0.0553
AQI_UCh-93384759	2376	2385	2383	2383	-

Number of samples used to estimate r^2^ are under the diagonal

Many genes usually affect quantitative traits and consequently the benefit of conducting MAS depends on the proportion of the variance explained by each QTL [44]. We found five markers significantly associated with resistance to *P. salmonis,* but the proportion of phenotypic variance and heritability explained are relatively small. In a typical GWAS analysis, markers need to surpass a specific threshold to be considered significant. In the case of traits that are affected by many genes of small effect, their effect can be underestimated [45]. On the other hand, polygenic approaches like genomic selection take into account the nature of such traits and include the information of all marker genotypes. Although there is no major effect QTL, the marker information can still be used for breeding programs [46] to predict the GEBVs of selection candidates [44] through GS. GS takes into account all markers to estimate the GEBVs, without the need to surpass a significance threshold for association with a particular trait [44]. The sib-test design can be used for MAS [47] or GS, where the association between markers and phenotypes is estimated in the sibs of the candidates, and the candidates are selected on breeding values that result from summing the estimates of the effects of their marker alleles [48]. Different approaches can be used to determine the marker effects to estimate the breeding values of the candidates. The GS methods facilitates computation of individual breeding values for all genotyped animals and do not require any prior knowledge of the underlying QTL [49]. Taking the relatively small proportion of the genetic variance explained by the significant markers into account, the results presented in this study suggest that a GS approach will be the most appropriate way of incorporating molecular information to assist artificial selection for *P. salmonis* resistance in Atlantic salmon.

## Conclusions

This is the first study designed to dissect the genetic basis for the resistance for *P. salmonis* using a dense SNP array in Atlantic salmon. Resistance to *P. salmonis* can be described as a moderate polygenic trait, as there are likely several loci involved but each with a small effect. Five SNPs could be identified as significantly associated with *P. salmonis* resistance. These markers explained a relatively small proportion of the variance for the trait.

## Availability of supporting data

SNP data from the 50 K Array used in this study have been deposited on SalmonDB database [38] [http://salmondb.cmm.uchile.cl/download/Array-Aquainnovo-UChile/]. Sequences flanking significant markers can be found as Additional file 1: Table S1.

## Additional file

Additional file 1: Table S1. Sequences flanking significant markers identified in GWAS. (DOCX 13 kb)

## Abbreviations

GWAS: Genome Wide Association Study
LD: Linkage disequilibrium
MAS: Marker assisted selection
QTL: Quantitative trait loci
SNP: Single nucleotide polymorphism
SRS: Salmon Rickettsial syndrome
GS: Genomic selection
