# Supplementary material for: Genome-wide association analysis reveals loci associated with resistance against Piscirickettsia salmonis in two Atlantic salmon (Salmo salar L.) chromosomes
Source: BMC Genomics. 2015 Oct 24;16:854. doi: 10.1186/s12864-015-2038-7 (PMC4619534; doi:10.1186/s12864-015-2038-7)
Supplement: Additional file 1: Table S1. — Sequences flanking significant markers identified in GWAS. (DOCX 13 kb) [file 12864_2015_2038_MOESM1_ESM.docx]

| Marker ID | Flanking Sequence |
| --- | --- |
| AQI_UCh-93346943 | ATTGGCTAGTTTCAGATATGCTGCGTAACTTTGCC[A/C]TGCCATTGAACCGTCTAGGTATTTAGAAAAGTAGA |
| AQI_UCh-93451692 | AAAATGGGAAGCAAAAAACAACTACTCCTGTCTTT[A/C]GACTATTGTCTACACATAATAGTTACAGGAAGAAC |
| AQI_UCh-93268476 | TCTCAGAGCCGCTTAACTATGTCACAAGGGAACGC[C/T]GGACTATCGCTTCTCAGGACGGATGAATAAAATGT |
| AQI_UCh-93323028 | TTCTTTACAAGACCTTCACCTTCTCTTTCACATTT[G/T]TTTTCACTTTATGTAGGCCTTTTGTACATGTGAAA |
| AQI_UCh-93384759 | AATCTCAATTGACTGTCAAGAAGACTGAAAAAATC[G/T]CAACATTTACAATTTCTAAATATGGACACAGTAAT |

**Additional file 1: Table S1.** Sequences flanking significant markers identified in GWAS.
